# Alcohol induced increases in sperm Histone H3 lysine 4 trimethylation correlate with increased placental CTCF occupancy and altered developmental programming

**DOI:** 10.1038/s41598-022-12188-3

**Published:** 2022-05-25

**Authors:** Yudhishtar S. Bedi, Haiqing Wang, Kara N. Thomas, Alison Basel, Julien Prunier, Claude Robert, Michael C. Golding

**Affiliations:** 1 Department of Veterinary Physiology & Pharmacology, College of Veterinary Medicine and Biomedical Sciences, Texas A&M University, 4466 TAMU, College Station, TX 77843 USA; 2 Genomics Center, Centre Hospitalier Universitaire de Québec–Université Laval Research Center, Quebec, QC Canada; 3 Département des Sciences Animales, Faculté des Sciences de l’agriculture et de l’alimentation, Université Laval, Québec, Canada

**Keywords:** Developmental biology, Embryology, Epigenetic memory, Epigenetics, Nuclear organization

## Abstract

Using a mouse model, studies by our group reveal that paternal preconception alcohol intake affects offspring fetal-placental growth, with long-lasting consequences on adult metabolism. Here, we tested the hypothesis that chronic preconception male alcohol exposure impacts histone enrichment in sperm and that these changes are associated with altered developmental programming in the placenta. Using chromatin immunoprecipitation, we find alcohol-induced increases in sperm histone H3 lysine 4 trimethylation (H3K4me3) that map to promoters and presumptive enhancer regions enriched in genes driving neurogenesis and craniofacial development. Given the colocalization of H3K4me3 with the chromatin binding factor CTCF across both sperm and embryos, we next examined CTCF localization in the placenta. We find global changes in CTCF binding within placentae derived from the male offspring of alcohol-exposed sires. Furthermore, altered CTCF localization correlates with dysregulated gene expression across multiple gene clusters; however, these transcriptional changes only occur in male offspring. Finally, we identified a correlation between genomic regions exhibiting alcohol-induced increases in sperm H3K4me3 and increased CTCF binding in male placentae. Collectively, our analysis demonstrates that the chromatin landscape of sperm is sensitive to chronic alcohol exposure and that a subset of these affected regions exhibits increased placental CTCF enrichment.

## Introduction

Our efforts to understand the effects of pre-and peri-conceptional exposures on the health of the growing fetus have almost exclusively focused on maternal stressors. However, recent studies show that paternal lifestyle and exposure history also exert pronounced effects on fetal-placental growth^1^. Moreover, in the offspring, these effects persist into adulthood and exert long-term impacts on inflammation, metabolic health, and disease emergence^2^. From these studies, we now realize that sperm carry an expansive suite of environmentally modifiable epigenetic marks, including DNA methylation, histone modifications, and small and long noncoding RNAs (ncRNAs) that significantly influence gene expression in the next generation^3^.

Due to the association with altered outcomes in offspring conceived using assisted reproductive technologies, most studies of epigenetic inheritance focus on DNA methylation and heritable changes in the regulation of imprinted genes^4^. Indeed, many studies examining paternal exposures to various nutritional, chemical, or drug-based stressors report corresponding changes in sperm DNA methylation profiles^3^. Similarly, sperm contain a diverse repertoire of ncRNAs that also change in response to various environmental stimuli and correlate with phenotypic changes in the offspring^5^. Significantly, injecting individual or specific subpopulations of ncRNAs into naive zygotes is sufficient to induce a subset of disease phenotypes in the next generation.

Although most nucleosomes are stripped from sperm and replaced with protamines^6^, mature sperm retain histones over ~ 5% of the genome, localizing to gene promoters, enhancers, and super-enhancer regions^7^. While less well studied than DNA methylation and ncRNAs, emerging evidence indicates that environmental stressors also alter the profile of sperm histone enrichment. For example, recent studies in rodents can correlate dietary-induced decreases in histone H3 lysine 9 dimethylation (H3K9me2) with adverse metabolic outcomes in the fetus^8,9^. Likewise, alterations in sperm histone H3 lysine 4 trimethylation (H3K4me3) induced by paternal folic acid deficiency correlate with defects in fetal-skeletal patterning^10^. Importantly, a subset of the alterations in sperm H3K4me3 persists in 8-cell embryos produced by these gametes, suggesting some diet-induced alterations either partially transmit or influence their abundance in the early conceptus.

However, following chromatin states through the transitions from sperm to the conceptus and discerning an impact on embryonic transcription is incredibly challenging, with some studies suggesting sperm chromatin organization is completely lost while others identify some conservation in the early zygote^11–22^. Therefore, despite a large body of research describing epigenetic inheritance through sperm, a direct link between sperm chromatin changes and impacts on the transcriptional programs driving early embryonic development and disease emergence remains obscure.

Rather than relying on any single feature, it is likely that multiple facets of sperm chromatin- and ncRNA-delivered mechanisms cumulatively mediate the intergenerational (or transgenerational) transmission of epigenetic memories. One of these multifaceted interactions potentially exists between H3K4me3 and the zinc finger protein CCCTC-Binding Factor (CTCF), which colocalize in both gametes and early embryonic cells^10,18,23^. CTCF and its interacting partner, the cohesin complex, organize and facilitate inter-loop bridges between genes and their respective enhancers^24–28^. Notably, recent evidence indicates that CTCF remains bound to DNA through the earliest stages of post-fertilization development and that together, CTCF and cohesin reinforce the emerging transcriptional programs driving development^20,29^. However, the role of CTCF in mediating altered developmental programming, the relationship with sperm H3K4me3, and the paternal epigenetic inheritance of disease remain poorly described.

Like other models of altered paternal epigenetic programming, preconception male ethanol exposures result in behavioral, growth, metabolic, and physiologic effects in the offspring^30^. Significantly, these phenotypes bear a striking similarity to clinical phenotypes described under fetal alcohol spectrum disorders (FASDs), indicating paternal alcohol use before conception is a significant contributing factor to the incidence and variation of this disorder. Further, they reveal that, in addition to acute toxicity, heritable alterations in the developmental program also drive the development of alcohol-induced behavioral and structural defects.

Using a physiologically relevant mouse model that simulates chronic binge drinking, our group has identified a correlation between preconception male alcohol exposures and defects in fetal-placental growth that precede long-term alterations in offspring health^31–36^. However, we observed minimal impacts of chronic alcohol use on sperm DNA methylation^32^. Further, we and the Homanic’s group separately identified alcohol-induced changes in sperm ncRNAs but with minimal overlap between candidate RNAs^31,37^. Using chromatin immunoprecipitation, we now address the hypothesis that alcohol alters the genomic enrichment of histone modifications in sperm by focusing on H3K4me3. We then assayed the gestational day 14.5 placenta, the phase wherein we can first detect deficits in placental gene expression and physiologic function, for alterations in CTCF localization. Finally, we examined the role of altered CTCF binding in disrupting placental gene expression and the correlations with H3K4me3 enrichment in sperm. This investigation reveals that, unlike DNA methylation, alcohol impacts sperm histone posttranslational modifications and that some of these changes may lead to long-lasting alterations in the developmental programs regulating placental function.

## Results

### Chronic alcohol exposure increases H3K4me3 levels at gene promoters and regulatory regions in sperm

Chronic folic acid deficiency alters the profile of sperm H3K4me3 enrichment, heritably impacting the distribution of this posttranslational modification in 8-cell embryos^10^. Therefore, we examined this environmentally-labile chromatin modification to determine if alcohol impacts H3K4me3 enrichment and distribution in sperm. To this end, we used our published Drinking in the Dark model to expose adult (postnatal day 90) C57BL/6 J males to either water or 10% ethanol (EtOH) for ten weeks. After verifying the impact of the preconception EtOH treatment on placental growth^36^, we euthanized the sires and generated sperm ChIP-seq libraries from control and EtOH-exposed males (n = 3 and 4), with an average of 50 million unique reads per sample.

**Figure 1:**
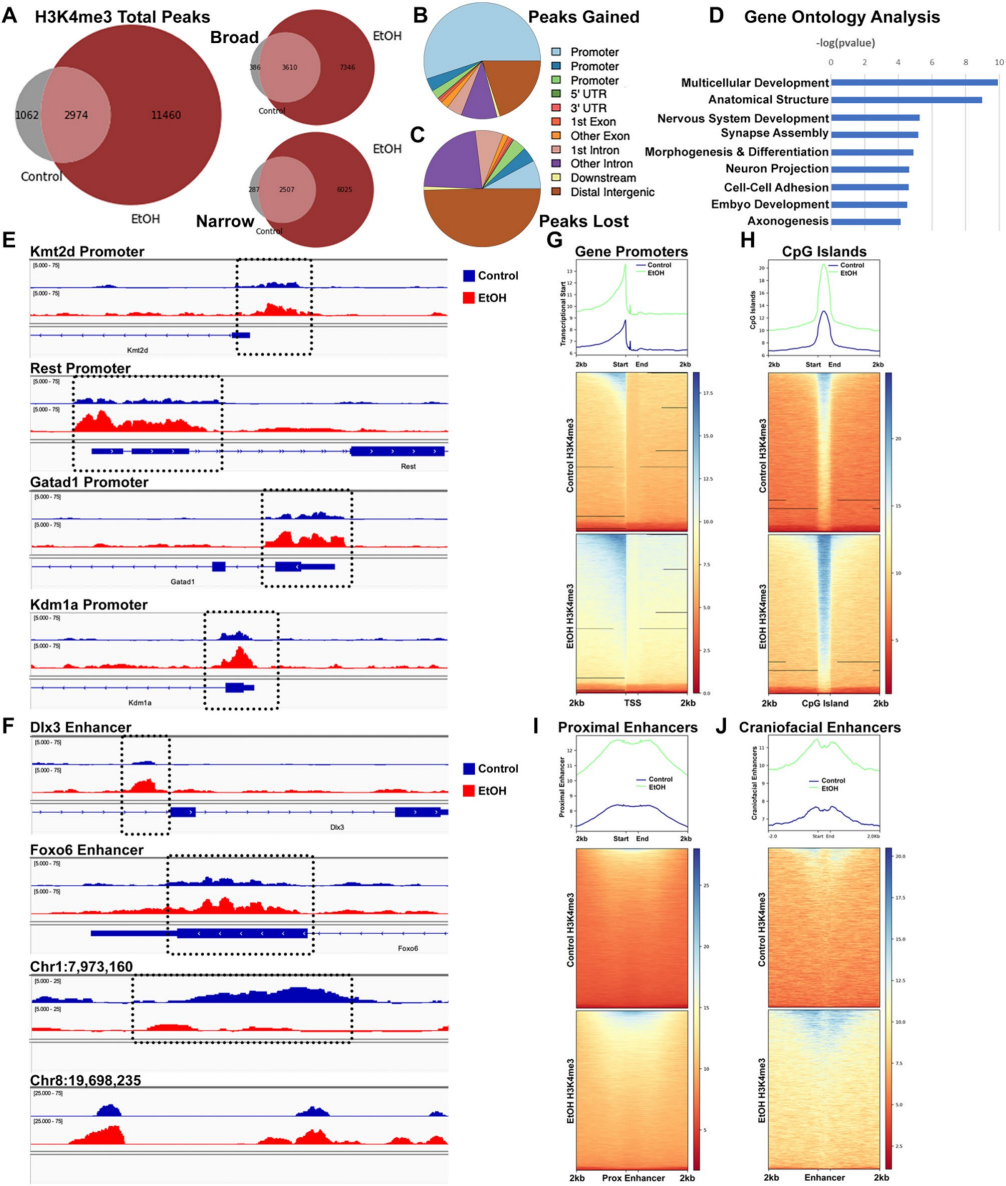
Chronic alcohol consumption increases H3K4me3 enrichment within the promoter and regulatory regions of critical developmental regulators. (**A**) Venn diagrams comparing total, broad, and narrow H3K4me3-enriched peaks between sperm isolated from control and EtOH-exposed males. Distribution of genomic regulatory features in (**B**) peaks gained and (**C**) lost in alcohol-exposed sperm. (**D**) Gene ontology analysis of peaks gained in alcohol-exposed sperm. Integrative Genome Viewer tracks showing altered H3K4me3 within the (**E**) promoter or (**F**) putative enhancer regions of genes involved in embryo development*.* Heatmaps displaying enrichment of H3K4me3 signals across (**G**) gene transcriptional start sites, (**H**) CpG islands, (**I**) ENCODE proximal enhancer elements^40^, and (**J**) gene enhancers regulating mouse craniofacial development^41^. We conducted ChIP-seq analysis on sperm isolated from 3 control and 4 EtOH-exposed males (n = 3, 4).

Comparing our H3K4me3 profiles to published sperm ChIP-seq datasets^7,10^, we identified similar enrichment profiles, predominantly mapping to gene promoter regions (Supplemental Fig. 1). Informatic analyses revealed that sperm derived from EtOH-treated males contained almost three times as many H3K4me3-enriched regions, with increases in both broad and narrow peaks (Fig. 1A). Novel peaks gained in sperm derived from EtOH-exposed males predominantly mapped to gene promoters (60%), with the remainder localizing to intronic (10%) and distal intergenic regions (20%) (Fig. 1B). Unexpectedly, the limited number of H3K4me3 peaks lost in EtOH-exposed sperm primarily mapped to distal intergenic and intronic regions (50 and 22%, respectively), while only 15% mapped to gene promoters (Fig. 1C). Gene Ontology Analysis^38,39^ of candidate regions exhibiting increased enrichment in EtOH-exposed sperm associated with processes related to neuronal development, including synapse assembly (*p*-value 6.56E-06), synapse organization (*p*-value 7.36E-05), axonogenesis (*p*-value 7.30E-05), and neuron differentiation (*p*-value 1.46E-04) (Fig. 1D).

We identified increased H3K4me3 enrichment within the regulatory regions of several critical developmental regulators, including *Gatad1*, *Kdm1a*, *Kmt2d*, and the transcriptional repressor *Rest* (Fig. 1E). Agreeing with recent studies by Lismer et al.^10^, we also identified altered H3K4me3 enrichment within presumptive genic and distal intergenic enhancers (Fig. 1F). As examples, regions within the gene bodies of transcription factors *Dlx3* and *Foxo6* showed increased H3K4me3 enrichment in EtOH-exposed sperm, while intergenic regions, as on chromosomes one and eight, exhibited regions with both gains and loss. Consistent with these observations, compared to control samples, sperm derived from EtOH-exposed males displayed increased H3Km4e3 enrichment at transcriptional start sites and CpG islands (Fig. 1G–H). Furthermore, we also identified increased colocalization at enhancer-like sequences recently identified by the ENCODE consortium^40^, as well as at a specific subset of enhancers driving craniofacial development in mice^41^ (Fig. 1I–J).

**Figure 2:**
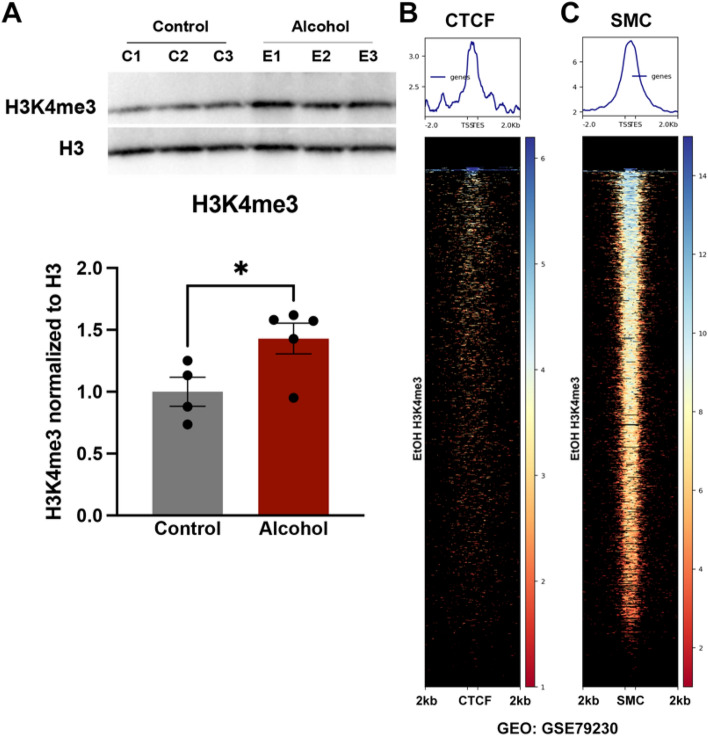
Increased H3K4me3 in sperm derived from EtOH-exposed males maps to CTCF and SMC-bound regions of the sperm genome. (**A**) Western blot analysis comparing total H3K4me3 normalized to histone H3 between preconception treatment groups (n = 4 control, 5 EtOH). Heatmaps displaying enrichment of EtOH-induced H3K4me3 signals across (**B**) CTCF- and (**C**) SMC-bound loci, previously identified in sperm (Jung et al. 2017, Geo: GSE72784).

To verify this overt increase in H3K4me3 enrichment, we acid extracted histones from the sperm of control and EtOH-exposed males and used western blotting. After normalizing to total histone H3, we confirmed an increase in H3K4me3 within the sperm of males chronically exposed to EtOH (Fig. 2A and Supplemental Fig. 2). Collectively, these results indicate that chronic exposure to EtOH increases H3K4me3 in sperm, affecting enrichment at gene promoters and presumptive enhancers associated with neural development and craniofacial patterning. Further, they indicate that increased H3K4me3 enrichment in sperm may serve as a biomarker of male alcohol exposure.

### EtOH H3K4me3-Enriched Regions Correlate with CTCF and cohesin-bound sites in sperm

In embryonic stem cells, H3K4me3 exhibits a prominent colocalization with the zinc finger protein CCCTC-Binding Factor (CTCF)^23^. Similar correlations exist between CTCF, cohesion, and H3K4me3-enriched loci across sperm and 2-cell embryos^10,18^, indicating these epigenetic factors may mutually support their intergenerational inheritance. Therefore, we compared H3K4me3-enriched narrow peaks in EtOH-exposed sperm to the published profiles of both CTCF and Structural Maintenance Of Chromosomes 2 (SMC2), a subunit of cohesin^7,18^. Our analyses identified enrichment of novel H3K4me3-enriched peaks over both CTCF- and SMC-bound regions in sperm (Fig. 2B–C). These observations suggest that chronic alcohol may influence the inheritance of H3K4me3, CTCF, or cohesin in the next generation.

### Preconception male alcohol exposure alters CTCF binding within placentae of the male offspring

Despite the colocalization of H3K4me3 and CTCF in gametes and early embryonic cells^10,18,23^, the role of CTCF in mediating alterations in developmental programming remains poorly described. Therefore, we set out to determine if altered H3K4me3 enrichment in sperm has a lasting impact on patterns of CTCF binding in the offspring. To address this question, we used chromatin immunoprecipitation followed by deep sequencing to assay enrichment of CTCF in the gestational day 14.5 placenta. This developmental stage represents the earliest phase in which we can detect alterations in placental efficiency and gene expression patterns^32^, and allows the investigation of epigenetic inheritance without the technical challenges of working with ultra-low-input samples. Given that placental growth phenotypes induced by paternal alcohol exposure are more severe in the male offspring^31,32,36^, we focused our analysis on male placentae.

**Figure 3:**
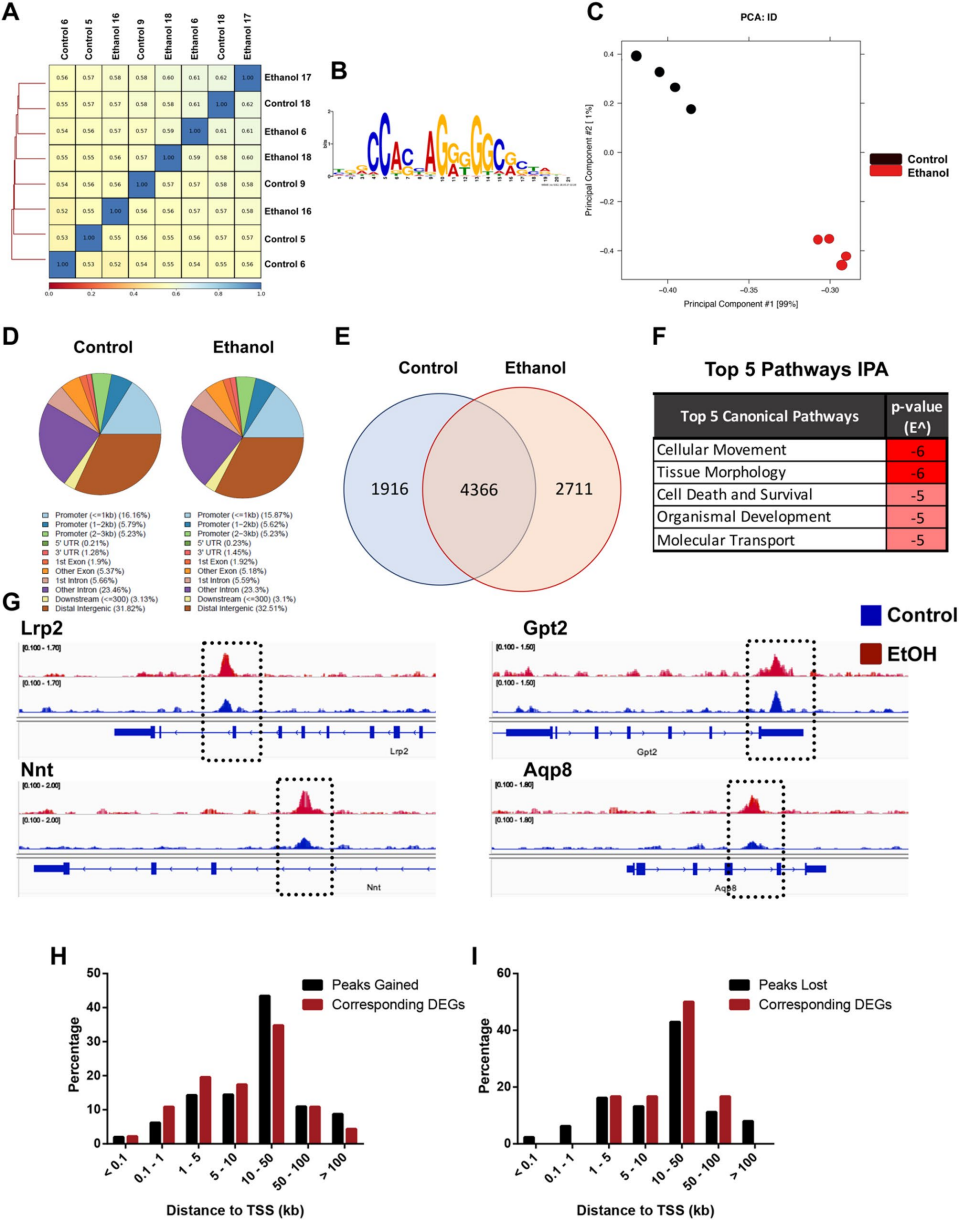
Chromatin immunoprecipitation analysis reveals preconception paternal alcohol exposure induces alterations in placental CTCF localization. (**A**) Correlation analysis between placentae derived from the male offspring of control and EtOH-exposed sires (n = 4). (**B**) Strong enrichment of the consensus CTCF binding site in precipitated DNA. (**C**) Principal component analysis and (**D**) genomic distribution of CTCF precipitated fragments compared between the preconception treatment groups. (**E**) Venn diagram and (**F**) Ingenuity Pathway Analysis comparing differential patterns of CTCF binding between male placentae derived from control and EtOH-exposed sires. (**G**) Integrative Genome Viewer tracks showing altered CTCF enrichment within the bodies of genes differentially expressed in placentae derived from the male offspring of EtOH-exposed sires^32^. Distribution of CTCF (**H**) peaks gained (**I**) and peaks lost with the transcriptional start sites of the differentially expressed genes.

Our sequencing experiments yielded a moderate correlation between experimental replicates (Fig. 3A). The identified peaks demonstrated strong enrichment of the consensus CTCF binding site (Fig. 3B; *p* = 1.1e–1146), and principal component analysis identified a clear separation between samples derived from the control and alcohol preconception treatment groups (Fig. 3C). Agreeing with previous studies examining CTCF localization^42^, ~ 30% of reads mapped to distal intergenic regions while the remainder mapped to gene bodies (~ 45%) and promoters (~ 25%), which was consistent between treatment groups (Fig. 3D). Using MACS2 with a minimal intersection of 100 bp, we identified 6282 high-confidence peaks in all samples derived from the offspring of control males and 7077 in offspring sired by alcohol-exposed males (Fig. 3E). Notably, 99% of identified peaks mapped to placental regulatory regions identified by the ENCODE consortium (ENCSR121NJX)^40^.

Of the high-confidence CTCF peaks we identified, 4366 were consistent between both treatment groups. However, compared to placental tissues isolated from the offspring of controls, the male offspring of alcohol-exposed sires lost 1916 peaks and gained 2711 unique peaks (Fig. 3E). When we mapped these peaks to the nearest gene and examined this list using integrated pathway analysis, we identified an enrichment of pathways participating in cellular movement, tissue morphology, and molecular transport (Fig. 3F).

We then reanalyzed the RNA-seq datasets we previously derived from these tissue samples^32^ (log2-fold change, adjusted *p*-value < 0.5) and then contrasted the ~ 497 differentially expressed transcripts with the differential patterns of CTCF binding. Of these differentially expressed genes (DEGs), 64 (13%) exhibited altered CTCF binding within 100 kb of their transcriptional start sites (TSS); 42 (8.4%) exhibited increased CTCF binding, and 22 (4.4%) displayed reductions (Fig. 3G). These data are consistent with recent studies examining CTCF loss of function, demonstrating that ~ 20% of differentially expressed genes are directly bound by this protein^29,43–45^. Interestingly, a higher proportion of DEGs corresponded to altered CTCF binding 10–50 kb away from the affected gene TSS (chi-square analysis of distributions; *p*-value < 0.0001; Fig. 3H–I). In addition, a small subset (14 of 497) of the differentially expressed genes also exhibited altered H3K4me3 enrichment in sperm (data not shown).

### Reductions in CTCF occupancy within candidate gene clusters correlate with changes in gene expression

**Figure 4:**
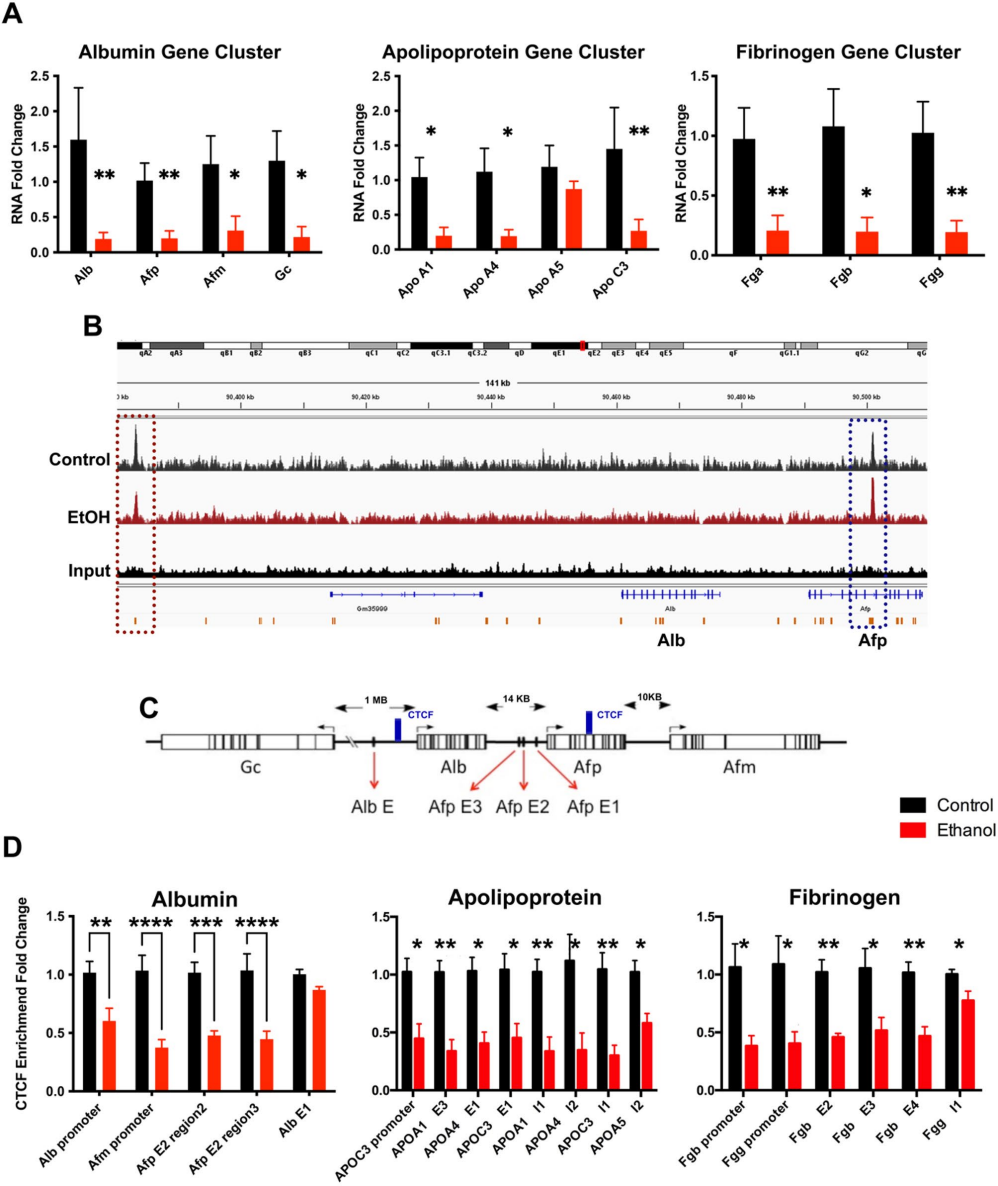
Preconception paternal alcohol exposure induces correlative patterns of altered gene expression and CTCF binding across the albumin, apolipoprotein, and fibrinogen gene clusters. (**A**) Using RT-qPCR and RNA samples isolated from male placentae derived from the offspring of EtOH-exposed sires, we assayed the expression of candidate genes within the *albumin*, *apolipoprotein*, and *fibrinogen* gene clusters. We normalized gene expression to the geometric mean of transcripts encoding *Sdha*, *Mrpl1*, and *Hprt* and examined eight placentae from each treatment, derived from five different litters (n = 8). (**B**) Integrative Genome Viewer tracks showing altered CTCF enrichment within the *albumin* gene cluster. (**C**) Map of the *albumin* gene cluster with known CTCF peaks previously identified in mouse liver^48^ demarcated in blue, and upstream enhancer regions identified by red arrows. Using qPCR, we measured CTCF enrichment within the *albumin*, *apolipoprotein*, and *fibrinogen* gene clusters. We assayed precipitated extracts for the enrichment of promoter regions, candidate Enhancers, or Insulators within the indicated loci. We performed ChIP experiments on eight placentae from each treatment, derived from five different litters (n = 8), and used a two-way ANOVA followed by an Uncorrected Fisher’s LSD. Error bars represent the standard error of the mean, *P < 0.05, **P < 0.01, ***P < 0.001, ****P < 0.0001.

In reexamining our RNA-seq datasets, we noted that several genes, colocalizing to the *albumin*, *apolipoprotein,* and *fibrinogen* gene clusters, all displayed coordinated reductions in gene expression. Using qRT-PCR, we confirmed reduced transcription of critical transport proteins in the *albumin* gene cluster, including *Alb*, *Afp*, *Afm*, and *Gc* (Fig. 4A). In addition to the albumin gene cluster, we observed downregulation of gene transcripts across both apolipoprotein gene clusters. These include genes in the Apoe/c1/c4/c2 gene cluster (chromosome 7, data not shown), and *Apoa1*, *Apoa4*, and *Apoc3*, which all localize to the gene cluster located on mouse chromosome 9 (Fig. 4A). In contrast, the lone remaining gene in this latter cluster, *Apoa5*, remained unchanged. Unlike the other genes in this cluster, *Apoa5* is not regulated by the centrally located *apoCIII* enhancer^46^. Finally, transcripts encoding the coregulated fibrinogen proteins *Fga*, *Fgb*, and *Fgg*, located in the *fibrinogen* gene cluster, were decreased in placentae derived from the male offspring of ethanol-exposed sires (Fig. 4A). Notably, except for *Apoa5*, the transcriptional dysregulation we observed impacted all the genes located in their respective clusters, with the entire complement of the *albumin*, *apolipoprotein*, and *fibrinogen* gene clusters demonstrating simultaneous down-regulation. These widespread reductions are similar to observations reported in experiments examining changes in gene expression induced by depletion of chromatin factors, including CTCF^24–28^.

Our ChIP-seq analysis identified altered CTCF enrichment at multiple loci across the differentially expressed gene clusters (representative peaks from the *albumin* gene cluster shown in Fig. 4B). Therefore, we focused on the *albumin, apolipoprotein*, and *fibrinogen* gene clusters to validate our ChIP-seq studies, as these regions all displayed altered CTCF binding and co-incident patterns of transcriptional dysregulation. Using qPCR, we assayed the enrichment of CTCF at select regions across these gene clusters (representative map of the *albumin* cluster is presented in Fig. 4C). We selected putative enhancers (E) and candidate insulators (I) based on the enrichment of CTCF from our datasets, recent studies of these gene clusters in the mouse and human liver^47–49^, as well as published profiles of the posttranslational histone modifications histone H3 lysine 27 acetylation (H3K27ac) and histone H3 lysine 4 monomethylation (H3K4me1) derived from CHIP-seq datasets in trophectoderm stem cells^50^. We identified altered enrichment of CTCF across multiple distal enhancer and promoter regions located within all three examined gene clusters (Fig. 4D). Notably, most of these changes localized to promoter regions or presumptive enhancers. Using the albumin cluster as an example, the large enhancer located upstream of the albumin gene^48^ is absent in placental cells. However, in the placenta, but not the liver, the region upstream of *alpha-fetoprotein* contains a prominent CTCF peak, which displays reduced binding in placentae derived from alcohol-exposed males (Fig. 4B, red box on the left). In contrast, the CTCF peak located within the gene body of *alpha-fetoprotein*, present in both liver and placental cells, increased in placentae derived from alcohol-exposed males (Fig. 4B, blue box, likelihood ratio of 2,829,863). These results indicate that select CTCF sites across these gene clusters display altered enrichment in placentae derived from the male offspring of ethanol-exposed males, which correlate with altered gene expression across the affected gene clusters.

### Alterations in placental CTCF occupancy do not correlate with changes in the cohesin complex member Rad21 or broad changes in enhancer-associated histone posttranslational modifications

CTCF and the cohesin complex are required for chromatin looping^44^, a subset of which facilitates promoter-enhancer interactions^29,51^. Importantly, cohesin lacks sequence-specific DNA binding activity and requires DNA binding factors, like CTCF, to be loaded onto chromatin^52^. Therefore, altered CTCF binding may influence both cohesin localization and enhancer function. Using ChIP, we examined placental extracts for the enrichment of cohesin complex member RAD21^27^. In addition, we also examined the enrichment of the posttranslational histone modifications histone H3, lysine four monomethylation (H3K4me1), and histone H3, lysine 27 acetylation (H3K27ac), given their association with poised and active gene enhancers, respectively^53^.

**Figure 5:**
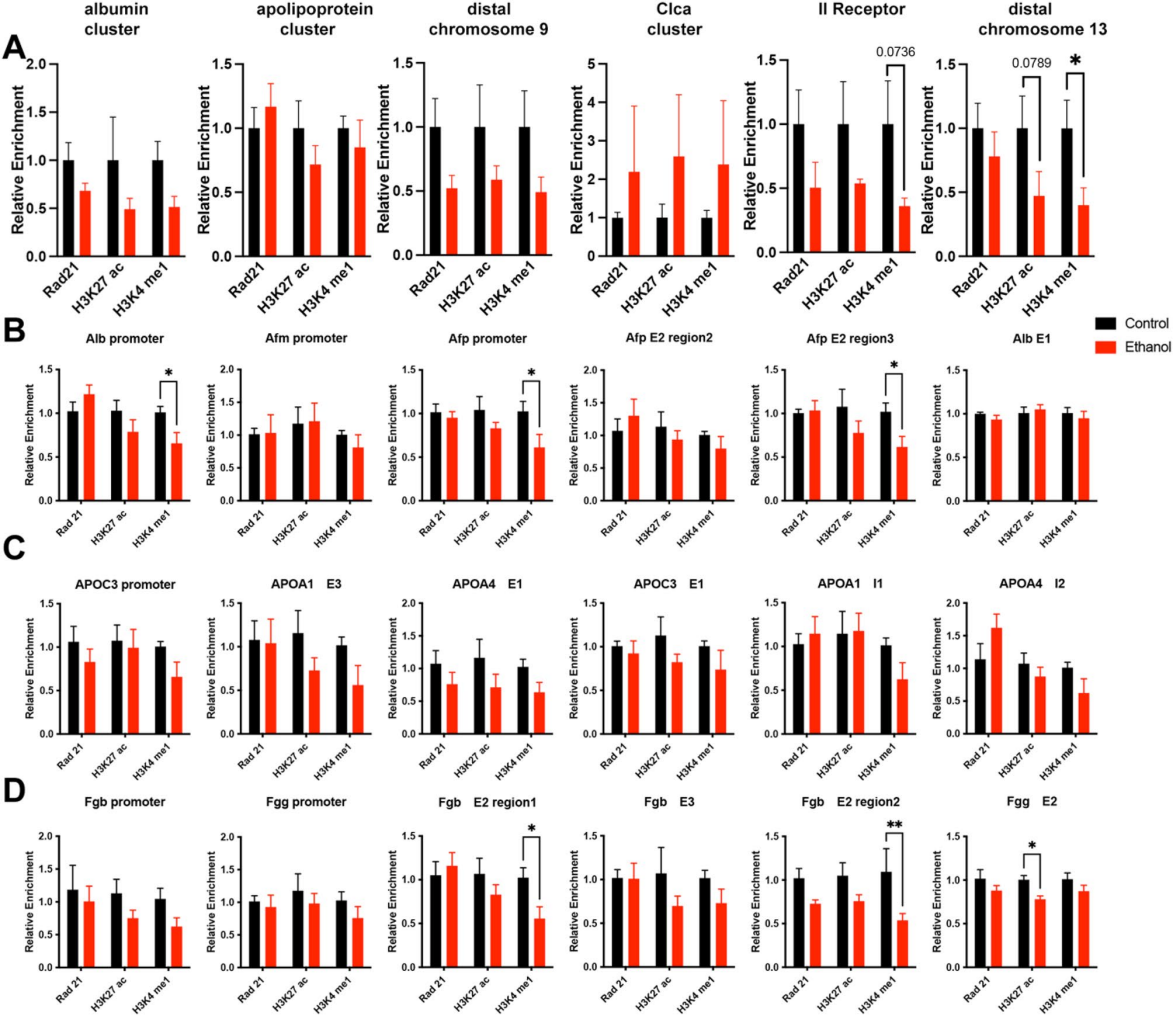
Consistent RAD21 binding and modest alterations in enhancer-associated histone posttranslational modifications in placentae derived from the male offspring of alcohol-exposed sires. We used ChIP qPCR to assay the enrichment of the cohesin component RAD21 and histone H3 lysine 27 acetylation (H3K27 ac) and lysine four mono-methylation (H3K4 me1) at (**A**) loci identified in our ChIP-seq analysis, as well as specific loci within the (**B**) *albumin*, (**C**) *apolipoprotein*, and (**D**) *fibrinogen* gene clusters identified in Fig. 4. We performed ChIP experiments on eight placentae from each treatment (n = 8), derived from five different litters, and used a two-way ANOVA followed by an Uncorrected Fisher’s LSD. Error bars represent the standard error of the mean, *P < 0.05, **P < 0.01.

Unexpectedly, using primers targeting the same candidate regions exhibiting altered CTCF enrichment identified above, we did not observe any alterations in RAD21 binding, which was constant across all examined loci (Fig. 5). Further, only one region displayed a decrease in H3K27ac enrichment, while we observed reduced H3K4me1 at six out of the 24 (25%) loci examined (Fig. 5). From these data, we conclude that the altered CTCF localization we observed at these loci does not influence RAD21 enrichment or consistently impact colocalizing posttranslational histone modifications.

### Changes in placental gene expression induced by preconception paternal alcohol exposure are sex-specific

**Figure 6:**
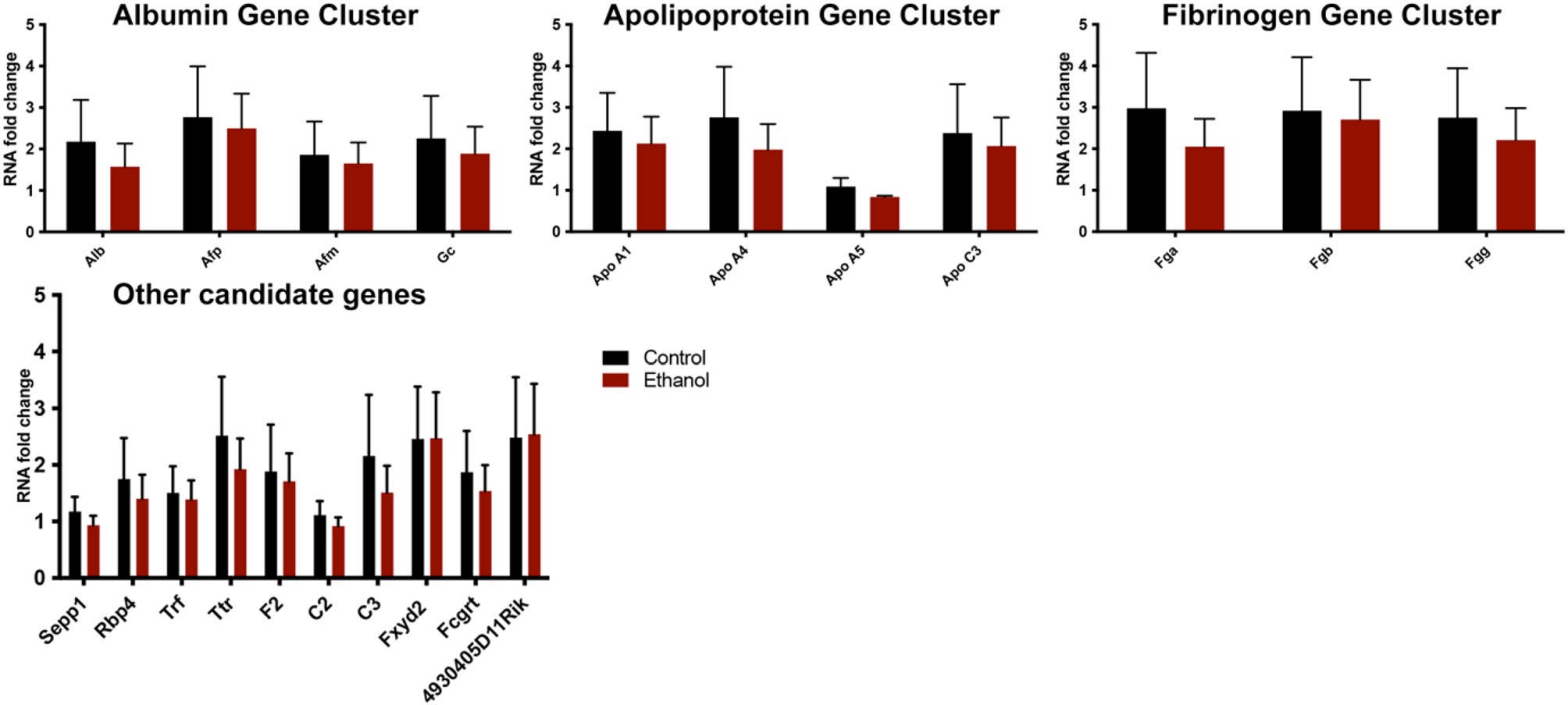
Placentae derived from the female offspring of EtOH-exposed sires do not exhibit altered gene expression of candidate genes identified in the male offspring. Using RT-qPCR and RNA samples isolated from female placentae derived from the offspring of EtOH-exposed sires, we assayed the expression of candidate genes within the *albumin*, *apolipoprotein*, and *fibrinogen* gene clusters, as well as other candidate genes identified in RNA-sequencing experiments. We normalized gene expression to the geometric mean of transcripts encoding *Sdha*, *Mrpl1*, and *Hprt* (n = 8) and compared treatments using a one-way ANOVA. Error bars represent the standard error of the mean.

Our previous studies of preconception paternal alcohol exposures have identified several sex-specific outcomes, including sexually dimorphic changes in gene expression and histology^31–33,36^. We, therefore, assayed the expression of genes within the *albumin*, *apolipoprotein*, and *fibrinogen* gene clusters, along with a selection of other candidate genes in placentae isolated from the female offspring of alcohol-exposed sires. We did not observe any significant differences in candidate gene expression in female placentae (Fig. 6). These observations reveal that the differences we observe across the gene clusters are specific to the male offspring of alcohol-exposed sires.

### Alcohol-induced gains in sperm H3K4me3 enrichment correlates with increased placental CTCF occupancy in male offspring

**Figure 7:**
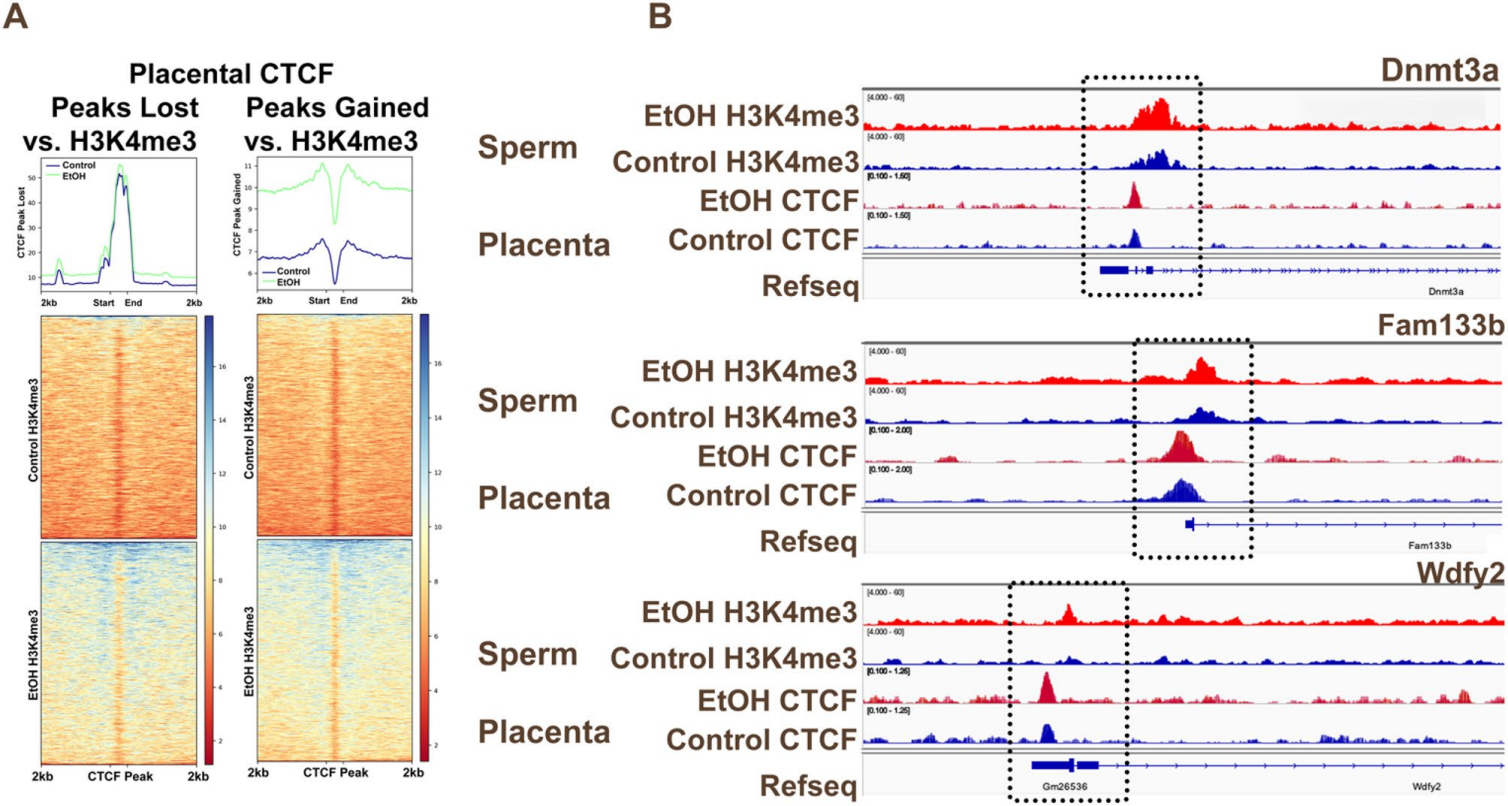
Correlation between EtOH-induced increases in sperm H3K4me3 enrichment and increased CTCF occupancy in male placentae sired by EtOH-exposed fathers. Heatmaps displaying H3K4me3 signals from sperm derived from control and EtOH-exposed males across (**A**) CTCF peaks lost and CTCF peaks gained in male placentae derived from the offspring of EtOH-exposed sires. (**B**) Integrative Genome Viewer tracks comparing altered H3K4me3 enrichment in alcohol-exposed sperm (top) with altered placental CTCF enrichment (bottom) across the promoter regions of *Dnmt3a, Fam133b,* and *Wdfy2*.

Finally, using deepTools2, we compared H3K4me3 enrichment in sperm with patterns of placental CTCF binding, separately examining peaks gained and peaks lost in the offspring of alcohol-exposed sires. Regions exhibiting a loss of placental CTCF exhibited H3K4me3 enrichment directly over the CTCF binding site. We found no differences when comparing H3K4me3 enrichment between sperm isolated from control and alcohol-exposed males (Fig. 7A). In contrast, sperm H3K4me3 was highly enriched 1 kb upstream and downstream of regions exhibiting increased CTCF binding in placentae isolated from the offspring of alcohol-exposed sires while notably depleted over the CTCF sites themselves. Moreover, regions exhibiting increased CTCF binding in placentae displayed increased H3K4me3 enrichment in alcohol-exposed sperm (Fig. 7A). These patterns were evident in the vicinity of the regulatory regions for the genes *Dnmt3a*, *Fam133b*, and *Wdfy2* (Fig. 7B). Our observations indicate that alcohol-induced increases in H3K4me3 enrichment in sperm may influence the inheritance of augmented CTCF enrichment in the placenta.

## Discussion

In the United States, seventy percent of all males report the consumption of alcohol, while forty percent drink at a binge level^54,55^. Using a physiologically relevant mouse model of male binge drinking, we tested the hypothesis that chronic preconception alcohol exposure impacts histone enrichment in sperm and that these changes are associated with altered developmental programming in the placenta. We find that alcohol-exposed sperm display increased H3K4me3 enrichment, localizing to specific genomic regulatory regions, including gene promoters and putative enhancers. Notably, some of these regions overlap with altered CTCF localization in placentae derived from the male offspring of alcohol-exposed sires. These results suggest a potential interaction between sperm H3K4me3 and CTCF in transmitting an epigenetic memory of paternal alcohol exposure to the offspring.

Although previous studies suggested chronic alcohol consumption inhibits one-carbon metabolism^56^, we do not observe any changes in s-adenosylmethionine or DNA methylation levels^32^, and here, we observed a generalized increase in sperm H3K4me3 enrichment. Similar to studies of folic acid deficiency^10^, the increased H3K4me3 we observed localized to CpG rich promoter regions, while areas with reduced enrichment predominantly mapped to intronic or distal intergenic regions. Areas displaying increased H3K4me3 enrichment mapped to the regulatory regions of genes involved in neurodevelopmental processes, while in contrast, we only identified 212 genes with decreased H3K4me3, not enriched in any specific biological pathways. Interestingly, similar to Lismer et al. we also identified altered H3K4me3 enrichment in craniofacial enhancers^10,41^. As craniofacial defects and CNS dysfunction are diagnostic criteria of fetal alcohol syndrome, an important next step will be determining if paternal drinking can induce facial dysmorphogenesis and CNS patterning defects in the offspring, similar to maternal alcohol exposures. Collectively, our analysis demonstrates that the chromatin landscape of sperm is sensitive to chronic alcohol exposure and that regions with increased or decreased H3K4me3 occur at distinct genomic loci. However, whether the alcohol-induced changes we observe in sperm result from deficiencies in histone removal^57^ or are part of a larger programmed response remains unknown and will be the subject of future investigation.

Previous studies examining chromatin structure in sperm, 2-cell embryos, and embryonic stem cells report overlapping patterns of CTCF, cohesin, and H3K4me3 enrichment^10,18,23^. These observations suggest CTCF, cohesin, and H3K4me3 may interact to transmit epigenetic information to the zygote, modulating early embryonic gene expression. However, some studies suggest that H3K4me3 peaks are depleted in zygotes^22^, while others indicate that some regions persist^10^. As CTCF enriched regions present in round spermatids and sperm persist in both 2-cell embryos and mESCs^7,18^, and CTCF binding exhibits high conservation across differentiating cells^58^, we focused on changes in CTCF enrichment during a developmental phase when we can identify molecular and pathophysiological changes in placental function. Genomic regions gaining increased CTCF binding in placentae sired by alcohol-exposed males exhibited increased H3K4me3 enrichment in sperm. Furthermore, these regions predominantly localize to areas flanking the CTCF binding site. Previous studies of CTCF enrichment in sperm identified a similar paradigm, with flanking regions enriched in monomethylated H3K4 but depleted of H3K4me3^7^. Therefore, alcohol-induced increases in H3K4me3 at these regions in sperm may heritably influence CTCF localization in the conceptus, altering the developmental program. Alternatively, we and others have identified alcohol-induced changes in sperm-inherited noncoding RNAs (ncRNAs)^31,37^, which recent studies indicate may play a role in the sequence-specific recruitment of CTCF^59^. Thus, alcohol disrupts two factors potentially influencing CTCF localization: H3K4me3 enrichment and the repertoire of ncRNAs. Therefore, it is tempting to speculate that these alcohol-induced changes alter CTCF localization during syngamy, imparting a legacy of altered gene expression, which persists through preimplantation development.

Consistent with recently published studies of the *albumin* cluster in mouse liver^48^, we were able to identify CTCF enrichment within the *Afp* gene body, which demonstrated a 37% increase in samples derived from the alcohol treatment group. However, the previously described CTCF peak located ~ 55 kb upstream of the *Alb* promoter in the liver was absent in the placenta, with the next closest peak ~ 75 kb upstream. This more upstream peak exhibited reduced enrichment in placentae derived from the offspring of alcohol-exposed sires. Our qPCR-based ChIP analysis also identified decreased CTCF enrichment at multiple gene promoters, including at sites across the *albumin*, *apolipoprotein,* and *fibrinogen* gene clusters. Significantly, using cultured human cells, researchers have demonstrated that depleting CTCF protein using siRNAs or deleting individual CTCF binding sites using Cas9 causes the organization of the *apolipoprotein* and *fibrinogen* clusters to collapse, reducing the expression across these clusters by half^47,49^. We observed similar reductions in CTCF enrichment and gene expression in placentae derived from alcohol-exposed sires. We suspect that similar to previous studies, reduced CTCF destabilizes Enhancer-Promoter interactions required to maintain appropriate gene expression patterns^29^. Further, reductions in these critical transport proteins may contribute to the growth deficits we observe in alcohol-exposed offspring^31–33,36^.

In previous studies, depletion of CTCF or cohesin did not lead to widespread changes in chromatin posttranslational modifications despite changes in gene transcription^43,44,60,61^. Similarly, we do not observe broad changes in histone posttranslational modifications across most loci. Further, although it is well established that CTCF and cohesin interact, they also have roles independent from each other^62,63^. Previous studies examining enforced degradation of CTCF reveal that its role in genome organization is predominantly confined to regions within TAD boundaries, whereas cohesin helps coordinate the extrusion of the overall loop domain^44,61^. It is interesting to note that alterations in CTCF localization did not impact the enrichment of RAD21 at any of the candidate promoters, which is consistent with CTCF-independent targeting of cohesin via transcription factors^62,63^. Cumulatively, our data indicate that, like the *protocadherin* gene cluster^64^, the *albumin*, *apolipoprotein,* and *fibrinogen* gene clusters exhibit examples of CTCF-independent cohesin localization. Finally, our data also reinforce the assertion that environmental exposures influence CTCF localization^45^.

Studies from our lab demonstrate that paternal drinking is associated with sex-specific patterns of growth restriction and metabolic dysfunction, with male offspring exhibiting more severe phenotypes^31–34,36^. These data contrast with mouse models of maternal alcohol exposure, reporting female offspring exhibit more severe fetal growth restriction^65^. The human literature is mixed but suggests greater dysmorphology and worse cognitive outcomes in females, but that FAS males are less likely to survive, and that prenatal alcohol exposure exerts sexually dimorphic changes on the male and female brain^66–70^. Here, the alterations in placental gene expression we identified at gestational day 14.5 also exhibited sexually dimorphic patterns, with none of the differentially expressed candidate genes identified in male offspring exhibiting alterations in female placentae. Researchers postulate that variances in patterns of fetal-placental growth between males and females underly sex-specific phenotypic differences in response to shared stressors^71,72^. Although most studies examining fetal outcomes do not stratify their data by sex, several studies have emerged describing the paternal epigenetic inheritance of sexually dimorphic phenotypes. How programmed changes in sperm persist through fertilization, resulting in sex-specific alterations in gene expression, remains an open question central to the field of developmental programming.

Although we used the same model of exposure, one limitation to our study is that the placental tissues we examined in this study were generated by a previous cohort of males^32^and not sired by the alcohol-exposed males we examined in this study. Another limitation is that we only identify correlations between CTCF enrichment and placental gene expression at a single time point and do not know if altered CTCF enrichment drives altered transcription or if these changes persist across other developmental time points. Additional studies are required to determine if the observed alterations in placental CTCF localization represent a causal memory, altering embryonic gene expression, or are merely another symptom of altered developmental programming.

Until recently, we did not anticipate that paternal lifestyle would impact developmental outcomes in the offspring. The basis of this blindspot was a misconception that sperm only contribute DNA and that the global reprogramming wave occurring post-fertilization limits the transfer of information from spermatozoon to the embryo. Consequently, the onus and etiology of FASDS have remained intensely focused on maternal exposures. Our data and others support an epigenetic effect of paternal alcohol consumption on offspring development and open important questions into how this memory transmits through sperm and the duration of the paternal insult before conception. Therefore, we must determine if alterations in placental CTCF localization represent causal changes in the developmental program or a deviation in developmental trajectory downstream of a larger cascade in response to prior developmental shifts.

## Materials and methods

### Animal studies and alcohol exposures

All experiments were conducted under AUPs 2014-0087 and 2017–0308, and approved by the Texas A&M University IACUC. All experiments were performed following IACUC guidelines and regulations. Here, we report our data per ARRIVE guidelines. In the outlined experiments, we used C57BL/6 J strain (RRID:IMSR_JAX:000664) mice, which we obtained and housed in the Texas A&M Institute for Genomic Medicine, fed a standard diet (catalog# 2019, Teklad Diets, Madison, WI, USA) and maintained on a 12-h light/dark cycle. On postnatal day 90, we individually caged males and provided them with limited access to the preconception treatments one hour after the initiation of their dark cycle and ceased treatments after four hours of exposure. During this time, we provided experimental males access to a solution of 10% (w⁄v) EtOH (catalog# E7023, Millipore-Sigma, St. Louis, MO, USA). We switched between two water bags to ensure identical handling conditions for control males. After each week, we recorded the weight of each mouse (g) and the amount of fluid consumed (g) and then calculated weekly fluid consumption as grams of fluid consumed per gram body weight (please see^36^ for EtOH consumption patterns and plasma alcohol levels of the male cohort). After 70 days of exposure, we paired treated C57BL/6 males with naïve, 18-week-old C57BL/6 J dams. Experimental males generated at least one litter, which we used to validate an impact on offspring fetal-placental growth^36^, before being sacrificed for sperm isolation.

### Mouse sperm isolation

After 15–20 weeks of exposure, we sacrificed male mice using standard CO2 asphyxiation followed by cervical dislocation. After dissection, we placed the entire portion of the cauda, plus approximately 1 cm of vas deferens, from each animal, separately into one well of a 12-well plate containing 1 mL of pre-warmed (37 °C) PBS. We then squeezed sperm out of the vas with forceps and made four or five incisions to the cauda epididymis to allow sperm to swim out. Next, we incubated plates at 37 °C for 30 min, collected sperm, and diluted a ten μL aliquot 1:50 in diH2O to count cells using a Neubauer chamber slide. We then washed the samples in PBS, incubated sperm in somatic cell lysis buffer (SCLB: 0.1% SDS, 0.5% Triton- X-100) for 30 min on ice followed by another wash in SCLB, then confirmed purity using microscopy. Finally, we centrifuged sperm at 3000 g for 5 min, washed samples in PBS, and either proceeded into the ChIP protocol or snap-froze the sperm pellets and stored them at -80 °C for acid extraction.

### Sperm histone acid extraction and western blotting

We acid extracted histones using methods previously described^73^. Briefly, we resuspended frozen sperm pellets in Nuclei Isolation Buffer-250 with 0.3% NP-40 (15 mM Tris–HCl (pH 7.5), 60 mM KCl, 15 mM NaCl, 5 mM MgCl2, 1 mM CaCl2, 250 mM Sucrose, 1 mM DTT, 10 mM sodium butyrate, and 1:100 Halt protease inhibitor (Cat# PI78437, Thermo Fisher Scientific, Pittsburgh, PA, USA)) and rotated them at 4 °C for 30 min. We verified sperm lysis using microscopy and then centrifuged samples at 600 g for five minutes. We washed the samples twice using Nuclei Isolation Buffer-250 with no NP40 and, after the second centrifugation, resuspended the pellet in five volumes of 0.2 M H2SO4, then rotated the samples overnight at 4 °C. We then centrifuged samples at 4000 × g for 4 min, transferred histone-enriched supernatant to new tubes, added Trichloroacetic Acid to a final concentration of 20% by volume, and then incubated samples for two hours on ice. We centrifuged samples at 10,000 × g for five minutes at 4 °C, discarded the supernatant, and resuspended the pellet in 1 mL cold acetone/0.1% HCl. We then washed the pellet twice with 100% acetone, air-dried the sample, and resuspended the pellet in water.

We separated acid extracted histones on 10% sodium dodecyl sulfate–polyacrylamide gels and transferred proteins to PVDF membranes. We used anti-H3K4me3 (catalog no. ab8580; RRID: AB_306649; Abcam, Cambridge, MA, USA) and antiH3 (catalog no. ab1791; RRID: AB_302613; Abcam) as primary antibodies, visualized blots using secondary antibodies conjugated to horseradish peroxidase (catalog no. sc-2004; RRID: AB_631746; Santa Cruz Biotechnology, Santa Cruz, CA, USA) and an enhanced chemiluminescence detection system (LI-COR, Lincoln, Nebraska USA).

### Isolaiton of gestational day 14.5 placentae and sex determination

In a previous study^32^, we isolated gestational day 14.5 placentas from control and ethanol-sired litters. These tissue samples were snap-frozen using liquid nitrogen and maintained at -80 Celcius. We isolated genomic DNA using the DNeasy Blood and Tissue Kit (catalog # 69504; Qiagen, Germantown MD, USA) and used PCR amplification of the *Zfy* and *Xist* genes to determine sex. Primers are listed in Supplementary Information Table 1.

### Chromatin immunoprecipitation (CHIP) analysis

#### Sperm

We conducted chromatin immunoprecipitation on mouse sperm using a previously published protocol^74^. We separately collected sperm from individual control and EtOH-exposed males and pre-treated with 50 μM DTT for two hours at room temperature to open sperm chromatin. Next, we treated decondensed sperm with 90 U of MNase (New England Biolabs, #M0247S) for 5 min at 37C. We then pre-cleared MNase-digested chromatin with blocked A/G Sepharose beads for one hour at 4 ﻿°C. For each ChIP reaction, we added 5 μg of ChIP validated anti-H3K4me3 antibody^75^ (catalog no. ab8580; RRID: AB_306649; Abcam, Cambridge, MA, USA) to the pre-cleared chromatin and incubated it overnight a 4 ﻿°C. The following day, we added 50 μL of blocked beads for four hours at 4﻿ °C. Next, we washed the beads and eluted DNA in elution buffer. Following RNase A and proteinase K treatment, we purified DNA using phenol/chloroform isolation and ethanol precipitation and resuspended the pellet in 40 μL ultrapure water. We then sent samples to Quick Biology (Pasadena, CA). Libraries generated using 10 ng of DNA were subjected to 150 bp paired-end sequencing, obtaining ~ 50 million reads per sample.

#### Placentas

We dispersed placentas into single-cell suspensions using 100 μm cell strainers (catalog # 352360; Corning Life Sciences, Corning, NY, USA), as described previously^76^. We then performed Chromatin Immunoprecipitations (ChIP) as previously described^34^. We purified eluted DNA using the Qiaquick PCR Cleanup kit (catalog # 28106; QIAGEN). The antibodies we used include: anti-H3K4me1 (catalog # 39297; RRID:AB_2615075; Active Motif, Carlsbad, CA, USA); anti-H3K27ac (catalog # 39133; RRID:AB_2561016; Active Motif); anti-CTCF (catalog # 07-729, RRID:AB_441965; Millipore-Sigma, Burlington, MA, USA); and anti-Rad 21 (catalog # ab992; RRID:AB_2176601; Abcam, Cambridge, MA, USA). We used all antibodies at one ug/reaction, including the negative control IgG (catalog # SC-2027; RRID: AB_737197; Santa Cruz Biosciences, Santa Cruz, CA, USA). After purification, we shipped a portion of the purified ChIP DNA samples to the Whitehead Genomic Services (Cambridge, MA), where 200–400 bp size selected DNA libraries were generated using the Swift 2S DNA Library prep kit (catalog # 44384; Swift Biosciences, Ann Arbor, MI, USA). The libraries were sequenced as single-end 50-bp reads with an average depth of 35 million reads per sample. Please note, due to challenges arising from Covid-19, we sequenced sperm and placental ChIP libraries at separate facilities. Finally, using qPCR, we assayed precipitated DNAs for relative enrichment using the Dynamo Flash supermix (catalog # F-415XL; Thermo Scientific) on a Bio-Rad CFX38 PCR system. Primer sequences are listed in Supplementary Information Table 1.

### Analysis of gene expression using reverse transcription quantitative polymerase chain reaction (RT-qPCR)

We isolated RNA from placental tissues using Trizol (catalog # 15596026; Thermo-Fisher, Waltham, MA, USA) and treated 1ug of RNA with DNAase I (catalog# AMPD1; Sigma, St. Louis MS, USA) according to the recommended protocols. We mixed isolated RNAs with 1µL 10 mM dNTP (catalog # 18427-013; Thermo-Fisher, Waltham, MA, USA), 1µL random hexamer oligonucleotides (catalog # 48190011; Thermo-Fisher), 11µL water and incubated at 70 °C for 5 min. After cooling on ice, we added SuperScriptII reaction buffer, DTT, and SuperScriptII, according to the SuperScriptII system (catalog # 18064-071; Thermo-Fisher). We then incubated samples at 25 °C for 5 min, 42 °C for 50 min, 45 °C for 20 min, 50 °C for 15 min and finally 70 °C for 5 min. We measured the relative abundance of individual genes using the Dynamo Flash master mix (catalog # F-415XL; Thermo Scientific), according to the manufacturer’s protocol, on a Bio-Rad CFX38 PCR system. Primers are listed in Supplementary Information Table 1.

### ChIP-sequencing data processing

We uploaded all sequence files to the Galaxy server^77^ (usegalaxy.org) for further processing. We then used Trimmomatic^78^ to trim FASTQ files for end quality and remove adapters. We then mapped trimmed reads to the mouse reference genome (mm10) using the Burrows-Wheeler Alignment tool BWA^79^, and used BAMTools filter^80^ to retain reads with a minimum MAPQ score of 20 for downstream analysis as BAM files. We then downloaded published ChIP-seq profiles for sperm H3K4me3, CTCF, and SMC from Jung et al. 2017 (Geo: GSE72784^7^) and Lismer et al. 2021 (Geo: GSE135678^10^) and processed reads using the same workflow.

#### Peak calling and differential peak analysis

We used MACS2^81^ to analyze ChIP-seq datasets and to call both broad and narrow peaks enriched against mapped reads obtained from Input files with an FDR threshold of 0.05. First, we determined the fragment size ‘d’ from alignment results using the MACS2 *predictd* tool, and we used this data as the extension size for the MACS2 callpeak tool, used lower and upper m-fold bounds of 5–50, and set the minimum FDR (q-value) cutoff for peak detection at 0.05. Next, we converted Bedgraph treatment file outputs to BigWig files for visualization in Integrative Genomics Viewer (IGV)^82^. Next, we extracted peaks from the narrow-peaks (CTCF only) and broad-peaks outputs common to all control and treatment samples using BEDTools^83^. We only considered intersecting peaks if at least a 0.25 fraction of the genomic intervals overlapped in both samples. We characterized the peaks unique to the Treatment group as "gained peaks", while those found only in Control samples were "lost peaks". We then used the bdgdiff function in MACS2 to identify differential peaks with a likelihood ratio > 1000. Finally, we used ChIPSeeker^84^ to annotate identified peaks and determine the distance to the nearest transcriptional start site (TSS). We pulled annotated BED files containing mouse gene transcriptional start sites, CpG Islands, distal enhancer-like sequences, and proximal enhancer-like sequences from the UCSC table browser. We obtained placental-specific C57BL6 enhancer sequences (ENCSR121NJX) from the ENCODE portal. We then used deepTools2^85^ to compare our datasets to these regulatory features.

#### DNA sequence motif analysis

We converted the annotated peaks from ChIPSeeker to fasta format using bedtools-getfastaBed^83^, removed duplicates using cd-hit-dup^86^, then analyzed these large nucleotide datasets for Motif discovery, enrichment analysis, and clustering using MEME-ChIP^87^. We then compared the resulting enriched motif sequences to known motifs in the HOCOMOCO Mouse motif database using the motif comparison tool Tomtom^88^.

#### Visualization of peaks and regions of interest

We converted all peak files to BigWig files and visualized the separate tracks in IGV. We then loaded the BED files marking genomic intervals of peaks gained or lost directly from Galaxy to IGV. Finally, we downloaded and visualized the published annotation BED file ENCSR121NJX demarcating known candidate cis-regulatory elements in the C57BL6 placenta, which we obtained from the ENCODE portal (https://www.encodeproject.org/)^40^.

### RNA-sequencing data processing

We isolated and sequenced total RNA from gestational day 14.5 fetal placentae previously^32^. Here, we reanalyzed these data using updated informatics methodologies. First, we uploaded all sequence files to the Galaxy server^77^ (usegalaxy.org) and used MultiQC to perform quality control on the raw paired-end files. We then used Trimmomatic^78^ to remove adaptor sequences and RNA STAR to map the reads onto the mouse (Mus musculus) reference genome (UCSC version GRCm39/mm39). We then used featureCounts^89^ to determine read abundance and annotated reads against M27 GTF (GENCODE, 2020) with a minimum mapping quality per read of 10. Finally, we used the DEseq2 function^90^ with default parameters on the resulting featureCounts files to identify candidate genes with greater than a log2-fold change in gene expression levels and an adjusted *p*-value (q-value) of less than 0.5.

### Data availability

We deposited the sequence data generated in this study in the GEO database under accession number GSE203189.

### Data treatment and statistical analysis

For the analysis of western blots, we quantified band intensities using the densitometry feature of ImageJ (RRID: SCR_003070; National Institutes of Health, Bethesda, MD, USA) and imported values into Excel. We then derived a ratio of H3K4me3 intensity divided by the intensity for total histone H3. Next, we imported this ratio into the statistical analysis program GraphPad (RRID: SCR_002798; GraphPad Software, Inc., La Jolla, CA, USA) and set statistical significance at alpha = 0.05. Finally, we verified all datasets for normality using a Shapiro–Wilk test, then compared normalized H3K4me3 between treatments using an unpaired student’s t-test.

For RT-qPCR analysis of gene expression, the replicate cycle threshold (Ct) values for each transcript were compiled and normalized to the geometric mean of the three reference genes including, *succinate dehydrogenase complex, subunit A* (*Sdha* NM_023281), *mitochondrial ribosomal protein L1* (*Mrpl1* NM_053158) and *hypoxanthine-phosphoribosyl transferase* (*Hprt* NM_013556)^91^. First, we calculated normalized expression levels using the ddCt method described previously^92^. We then transferred the derived relative fold change values from each biological replicate into the statistical analysis program GraphPad, where we then verified data for normality using a Brown-Forsythe test. We set significance at *p*-value < 0.05 and utilized a one-way analysis of variance (ANOVA), followed by Tukey’s HSD analysis. In all instances, we have marked statistically significant differences with an asterisk.

For qPCR-based analysis of candidate gene regulatory region enrichment, we first normalized ChIP samples to 1% input, using the formula previously described^93^. Next, to independently examine alterations in each posttranslational modification, we normalized the means from each independent sample to the control average. We then tabulated the results of 3 independent experiments and calculated the cumulative means and standard error of the mean. Finally, we transferred the Values from each biological replicate into the statistical analysis program GraphPad. We verified normality using a Brown-Forsythe test and then an ANOVA to assess differences, followed by an Uncorrected Fisher’s LSD.

## Supplementary Information

Supplementary Information 1.

Supplementary Information 2.
