# Supplementary material for: Alcohol induced increases in sperm Histone H3 lysine 4 trimethylation correlate with increased placental CTCF occupancy and altered developmental programming
Source: Sci Rep. 2022 May 25;12:8839. doi: 10.1038/s41598-022-12188-3 (PMC9130987; doi:10.1038/s41598-022-12188-3)
Supplement: Supplementary file 2 — Supplementary Information 2. [file 41598_2022_12188_MOESM2_ESM.docx]

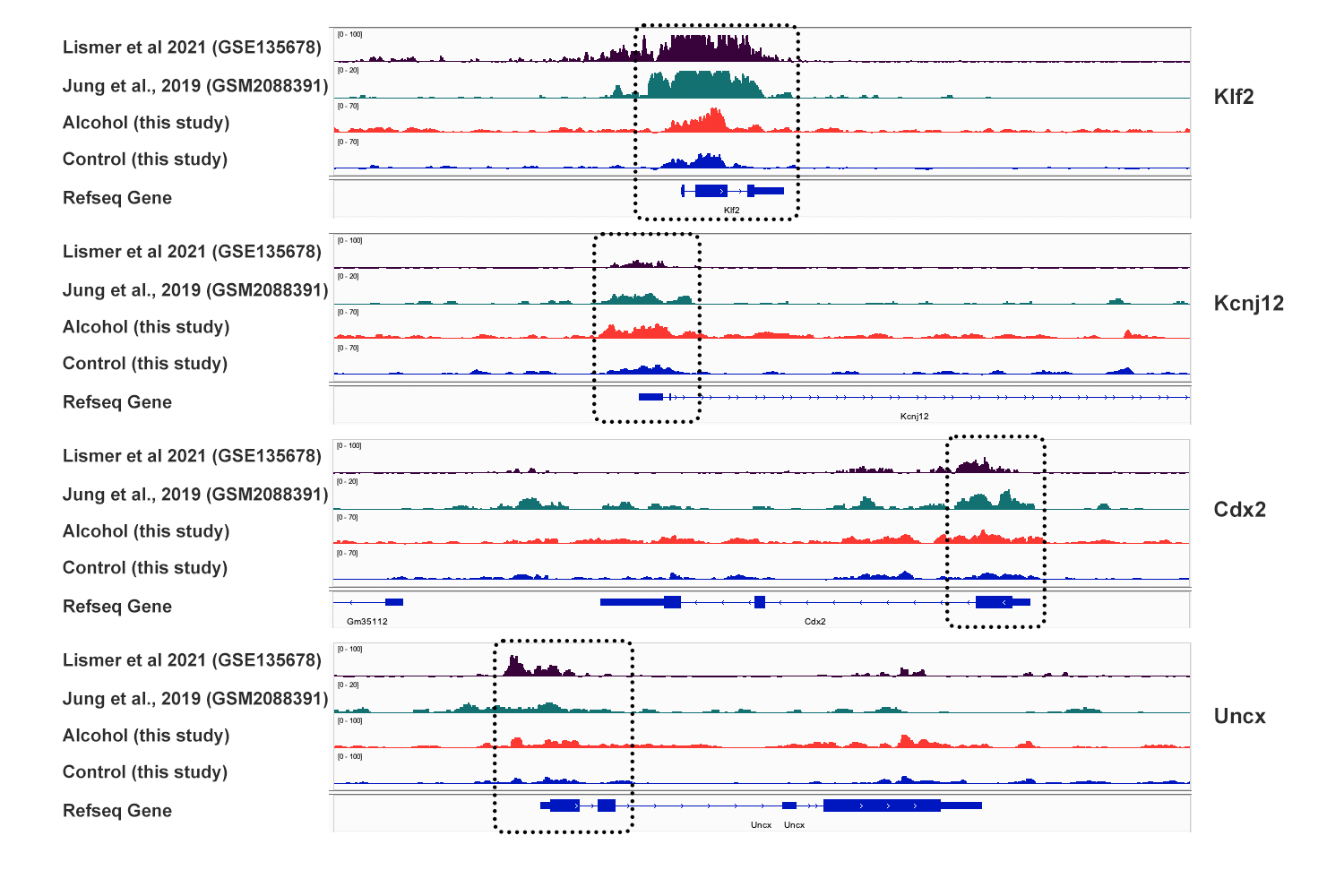


**Supplemental Figure 1. Comparison of published H3K4me3 ChIP-sequencing datasets to data generated in this study.** Integrative Genome Viewer tracks comparing histone H3 lysine 4 trimethylation (H3K4me3) enrichment in sperm between datasets generated by Jung et al., 2017 (Geo:GSE72784,^7^), Lismer et al., 2021 (Geo: GSE135678, ^10^) and this study.

**
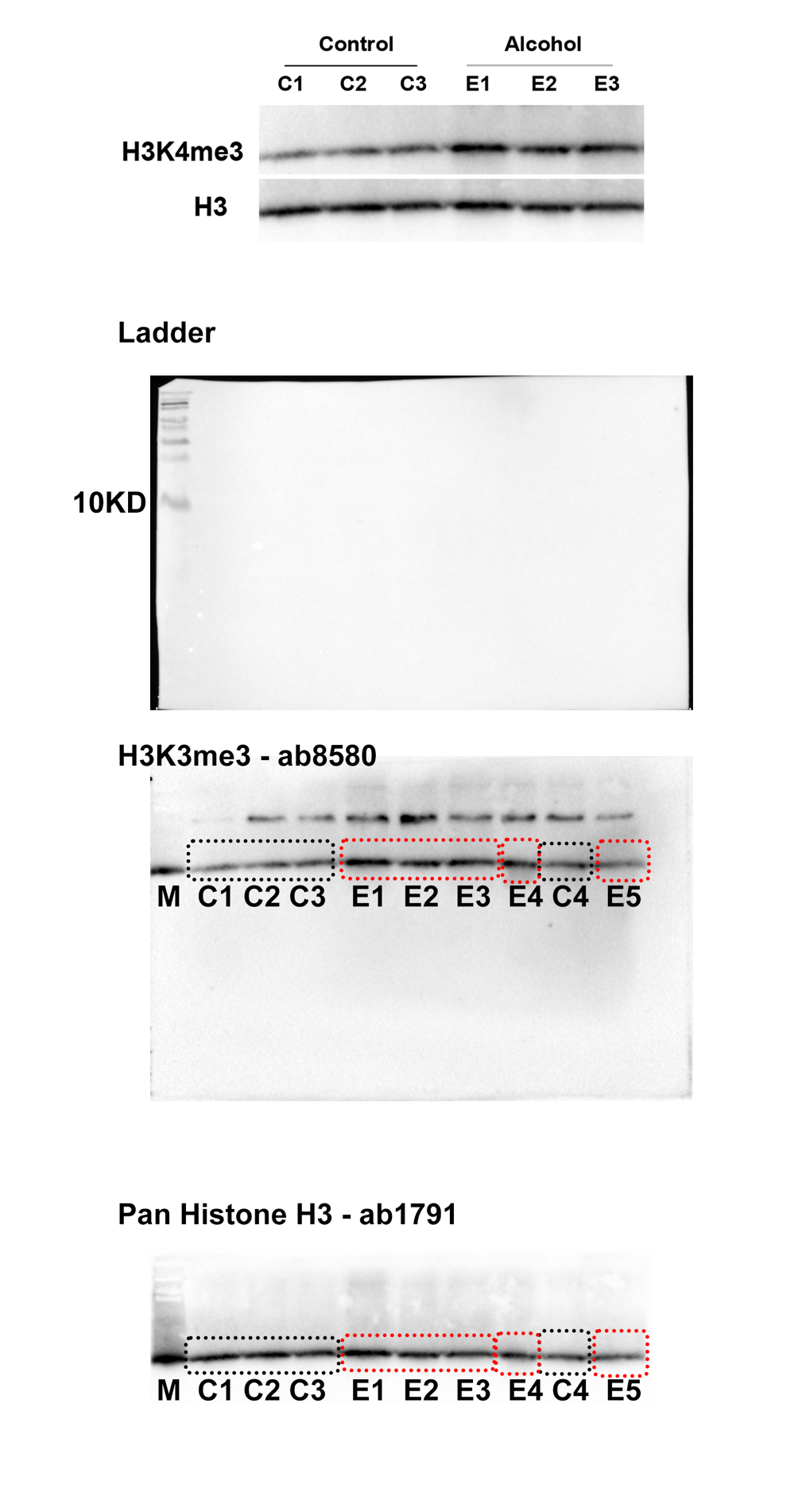
**

**Supplemental Figure 2. Full-length gels and blots used to generate the images presented in Figure 2a.**
